# Enhancing the REMBRANDT MRI collection with expert segmentation labels and quantitative radiomic features

**DOI:** 10.1038/s41597-022-01415-1

**Published:** 2022-06-14

**Authors:** Anousheh Sayah, Camelia Bencheqroun, Krithika Bhuvaneshwar, Anas Belouali, Spyridon Bakas, Chiharu Sako, Christos Davatzikos, Adil Alaoui, Subha Madhavan, Yuriy Gusev

**Affiliations:** 1 Medstar Georgetown University Hospital, Washington, DC USA; 2 Innovation Center for Biomedical Informatics (ICBI), Georgetown University, Washington, DC USA; 3 Center for Biomedical Image Computing and Analytics (CBICA), University of Pennsylvania, Philadelphia, PA USA

**Keywords:** Data publication and archiving, Data processing, Image processing

## Abstract

Malignancy of the brain and CNS is unfortunately a common diagnosis. A large subset of these lesions tends to be high grade tumors which portend poor prognoses and low survival rates, and are estimated to be the tenth leading cause of death worldwide. The complex nature of the brain tissue environment in which these lesions arise offers a rich opportunity for translational research. Magnetic Resonance Imaging (MRI) can provide a comprehensive view of the abnormal regions in the brain, therefore, its applications in the translational brain cancer research is considered essential for the diagnosis and monitoring of disease. Recent years has seen rapid growth in the field of *radiogenomics*, especially in cancer, and scientists have been able to successfully integrate the quantitative data extracted from medical images (also known as radiomics) with genomics to answer new and clinically relevant questions. In this paper, we took raw MRI scans from the REMBRANDT data collection from public domain, and performed volumetric segmentation to identify subregions of the brain. Radiomic features were then extracted to represent the MRIs in a quantitative yet summarized format. This resulting dataset now enables further biomedical and integrative data analysis, and is being made public via the NeuroImaging Tools & Resources Collaboratory (NITRC) repository (https://www.nitrc.org/projects/rembrandt_brain/).

## Abstract (summary)

**Table:** 

Measurement(s)	MRI scans
Technology Type(s)	Segmented labels in NIFTI format
Sample Characteristic - Organism	Homo sapiens

## Introduction

Brain cancer is a deadly disease with a 5-year survival rate of only about 30% (www.seer.cancer.gov). According to the Global Cancer Observatory https://gco.iarc.fr/, there were 308,102 cases of cancers of the brain and the central nervous system (CNS) in the world as of 2020^1^ (139,756 were women, and over 168,346 were men^1^). There are more than 120 identified types of brain tumors, according to the National Brain Tumor Society, that are extremely heterogenous in nature, https://braintumor.org/brain-tumor-information/understanding-brain-tumors/tumor-types/ making it a complex disease to understand and interpret. In spite of the progress made in treatments of other cancers over the last 20 years, there continue to be only 5 approved drugs to treat brain tumors, and no prognostic advancements for GBM patients have been observed^2^. https://braintumor.org/brain-tumor-information/brain-tumor-facts/.

Medical imaging technologies including magnetic resonance imaging (MRI) and computed tomography (CT) scans, are one of newer technologies increasingly used in translational imaging research^3^. Due to its complex nature, the brain tissue environment offers a rich opportunity for translational research. MRI can provide a comprehensive view of the abnormal regions in the brain^4^ therefore, its applications in the translational brain cancer research is considered essential for the diagnosis, monitoring, and management of the disease^3^.

In recent years, scientists have been able to integrate the data gleaned from medical images with genomics, and this burgeoning field is called *radiogenomics*^5–7^. The imaging data is first converted into a quantitative summarized format, through extracted measurements (also known as radiomics) that can be both visual and sub-visual to the naked eye^8^. These radiomic features allow further extraction of imaging phenotypes, that can be integrated with genomics data using machine learning (ML) and artificial intelligence (AI) based algorithms. While many clinical trials are ongoing for new treatments in brain cancer research, there are many opportunities for the development novel treatment hypotheses using radiogenomics approaches^9^.

There are several large-scale national collaborations that utilize either brain cancer data, or medical imaging related technologies for translational research including, the Brain Science Foundation https://www.brainsciencefoundation.org/; The *endbraincancer* (EBC) https://endbraincancer.org/end-brain-cancer/; The Children Brain Tumor Tissue Consortium (*CBTTC*) https://www.chop.edu/clinical-trial/cbttc-collection-protocol; The Children’s Brain Tumor Network https://cbtn.org/about-us, The Cancer Imaging Archive (TCIA)^10^, and more. However, only a handful of national brain cancer projects include both multi-omics data and medical imaging data. These include The Cancer Genome Atlas (TCGA), which is a large collection of multi-omics data from 22 cancer types including Lower grade gliomas (LGG)^11,12^ and Glioblastomas (GBM)^12,13^. The imaging data from the TCGA data collection, along with imaging data from other studies are housed at the publicly accessible TCIA imaging data repository https://www.cancerimagingarchive.net/. The National Cancer Institute (NCI) Cancer Research Data Commons (CRDC) provides access to a cloud-based ecosystem with access, visualization, and analysis of multi-modal imaging data through its public portal. It also allows researchers to connect imaging data to corresponding genomics and proteomics data within the CRDC collections https://portal.imaging.datacommons.cancer.gov/.

Another initiative that included both omics data and medical images was the REMBRANDT project (REpository for Molecular BRAin Neoplasia DaTa), a joint initiative of the NCI and National Institute of Neurological Disorders and Stroke (NINDS). This project consisted of a large brain cancer patient-derived dataset that contained clinically annotated data generated through the Glioma Molecular Diagnostic Initiative (GDMI) from 874 glioma specimens comprising 566 gene expression arrays, 834 copy number arrays, and 13,472 clinical phenotype data points. In 2015, the molecular data including microarray gene expression, copy number, and clinical data were migrated to the Georgetown Database of Cancer (G-DOC)^14,15^. This project was managed by our team at Georgetown University, and this dataset was made public in 2018 through the publication *Gusev et al*.^16^, and the data made available via the NCBI Gene Expression Omnibus (GEO) data repository GSE108476^17^. Among the patients in this REMBRANDT collection, pre-surgical magnetic resonance (MR) multi-sequence images was obtained from 130 patients and is hosted at TCIA^18^ https://wiki.cancerimagingarchive.net/display/Public/REMBRANDT.

In this paper, we obtained the raw MRI scans from the publicly available REMBRANDT collection, and processed them through a well-known image processing pipeline that is specialized for the brain cancer MRI scans. The workflow included automated volumetric segmentation of the MRIs that identified various subregions of the brain including necrotic core, edema, non-enhancing tumor (NET) and enhancing tumor (ET), Gray Matter (GM), White Matter (WM), and Cerebrospinal Fluid (CSF). A Board-Certified radiologist then performed verification and refinements of the segmented labels that included extracted radiomic features as well. This allowed the representation of the MRI scans in a quantitative format, with the intention of enabling further biomedical and integrative data analyses.

This dataset is being made public in the NeuroImaging Tools & Resources Collaboratory (NITRC) repository through this link (https://www.nitrc.org/projects/rembrandt_brain/)^19^ to allow researchers perform radiogenomics based analysis, integrate with gene expression and copy number data, and enable new discoveries and hypotheses. Table 1 shows a summary of the REMBRANDT brain cancer collection.

**Table 1:** Details of the REMBRANDT brain cancer collection.

Source	Protocol 1	Samples	Protocol 2	Data
Rembrandt glioma samples	RNA extraction	671 patients	Microarray hybridization	GSE108474^17^
Rembrandt glioma samples	DNA extraction	263 patients	SNP array hybridization	GSE108475^17^
Rembrandt glioma samples	MRI scans	130 patients	Raw MRIs in DICOM format	TCIA^18^
Rembrandt glioma samples	MRI scans	64 patients	Segmented labels in NIFTI format	NITRC^19^

## Materials and Methods

### Data download

We first downloaded the pre-operative raw MRI scans from the TCIA imaging archive^10,20^ for all the 130 patients including multiple series for each patient in DICOM file format^21^. The board-certified radiologist performed labeling of the MRI scans of the all modalities in the dataset that included MRIs from different modalities, including T1-weighted, T2-weighted, post-contrast T1-weighted (T1-C), and T2 Fluid-Attenuated Inversion Recovery (FLAIR) volumes^22^.

### Data formatting

**Fig. 1:**
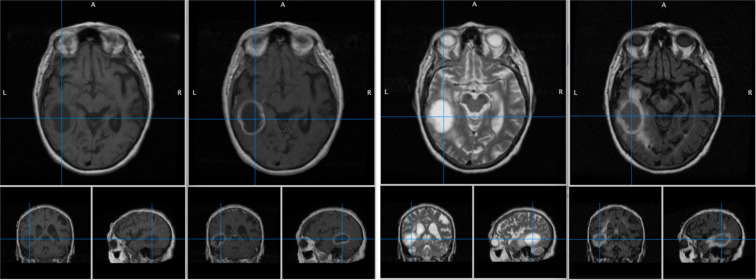
An example of four modalities (T1-weighted, T2-weighted, post-contrast T1-weighted (T1-C), and FLAIR) from the same brain cancer patient (patient# HF1702).

Some scans had mixed PD and T2 modalities, and had to be separated based on the meta-data in the DICOM file. Only patients that had available MRI data for all four modalities (T1, T2, T1-C and FLAIR) were selected for the next step, which resulted in a set of 72 patients. Figure 1 shows an example of four modalities from the same brain cancer patient.

**Fig. 2:**
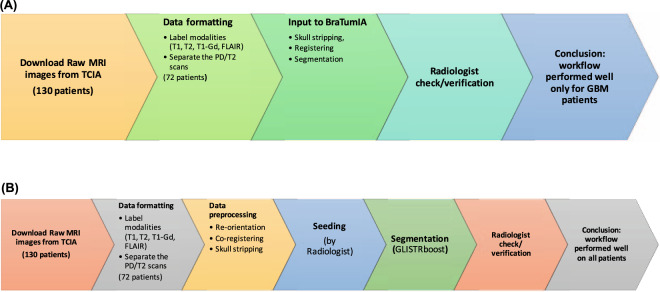
(**A**) Segmentation pipeline using the Bratumia segmentation tool. (**B**) Segmentation pipeline using the GLISTRboost segmentation tool.

We then applied two different pipelines for the processing of these scans, comprising two popular brain cancer segmentation tools: (a) The first pipeline used the BraTumIA^23^ tool (Fig. 2A), and (b) the second pipeline used the GLISTRboost^24,25^ tool (Fig. 2B). Notably, the GLISTRboost based pipeline was top ranked in the International Multimodal Brain Tumor Segmentation challenge 2015 (BraTS’15)^26^ and uses an Expectation-Maximization (EM)^27^ framework to automatically map the various sub-regions of the brain scans while accounting for brain deformations caused by the tumor through biophysical growth modelling^28^. The runner-up for this challenge was the BraTumIA tool which uses a machine learning algorithm^23^.

### Brain tumor segmentation using BraTumIA

After the raw data was downloaded and formatted, we ended up with MRI scans from 72 patients with four modalities - T1-weighted, T2-weighted, T1-C, and FLAIR. The images were then used as input into the BraTumIA^23^ tool which internally performed all processing steps. Skull stripping was performed using the Insight Toolkit ITK^29^ as a first step to generate a brain mask, and in the second step, the images were registered i.e. spatially transformed using the ITK toolkit, so that the voxels of the various images will correspond to one another. The images were segmented into tumor and healthy images using a joint classification-regularization based algorithm. The segmented output labels were in a meta image format (.mha) file format (Fig. 2A).

**Fig. 3:**
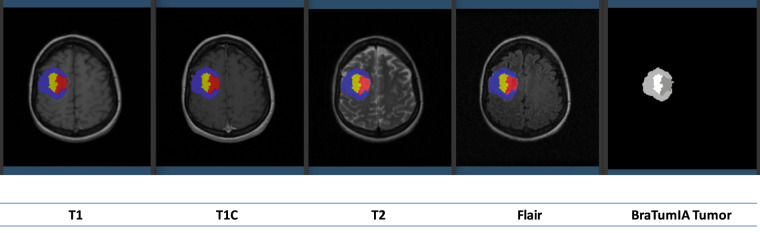
Segmented labels for a brain cancer patient (patient# HF1708) obtained using the BraTumIA pipeline. It shows how the MRI scans look across all four modalities.

The Board-Certified radiologist performed verification of the predicted segmented labels. Example segmented labels for a brain cancer patient obtained using the BraTumIA pipeline is shown in Fig. 3

### Brain tumor segmentation using GLISTRboost

The raw data was downloaded and cleaned in a similar order as the previous pipeline to get MRI scans from 72 patients with four modalities - T1-weighted, T2-weighted, T1-C, and FLAIR. Then, several pre-processing steps were applied. The MRI scans were first *re-oriented* so that all the images would be transformed into the same Left-Post-Superior (LPS) coordinate system https://www.slicer.org/wiki/Coordinate_systems, a necessary step in order to be able to compare or integrate data obtained from different modalities. The images were then *co-registered* to the same T1 anatomic template using “Greedy” (github.com/pyushkevich/greedy)^30^, a CPU-based C++ implementation of the greedy diffeomorphic registration algorithm^31^. Greedy is integrated into the ITK-SNAP (itksnap.org) segmentation software^32,33^, as well as the Cancer Imaging Phenomics Toolkit (CaPTk - www.cbica.upenn.edu/captk)^34–37^. After the co-registration, brain extraction (also known as skull-stripping) was performed using the Brain Mask Generator (BrainMaGe)^38,39^, which is based on a deep learning segmentation architecture (namely U-Net^40^) and uses a novel framework introducing the brain’s shape as a prior and hence allowing it to be agnostic to the input MRI sequence. BrainMaGe^38,39^ was used to remove non-cerebral tissues like the skull, scalp, and dura from brain images.

A step called *seeding* was then performed by the radiologist. Seeding involved manual tagging of the sub-regions of the brain MRI including tumor regions namely ET, NET and ED; and healthy regions including white matter, gray matter, CSF, vessels and cerebellum. Seed points included center and radius of the tumor, and sample seed points in each sub-region of the brain image. This seeding step enabled the segmentation algorithm to accurately model the intensity distribution (mean and variance), for each tissue class. This allowed the segmentation tool to perform with higher accuracy compared to other segmentation tools. This step was performed using the Cancer Imaging Phenomics Toolkit (CaPTk) software platform^34–37^. The output of this step included two text files - one with information about the tumor, and another regarding the sample points in each sub-region. These two files were used as input to the next step in the pipeline.

After these steps were completed, automated volumetric segmentation and registration was performed using GLISTRboost^24,25^. During the segmentation process, MRI scans from 8 patients had to be filtered out for several reasons including low quality and very limited coverage, or unreliable results due to irregularities in the input MRI scans. At the end of this pipeline (Fig. 2B), complete segmentation results were successfully obtained for 64 patients. Table 2 shows a summary of the original 130 patients in the REMBRANDT patient cohort before start of analysis, and the 64-patient cohort after completion of the segmentation step.

**Table 2:** Summary of the patient cohort in the REMBRANDT brain cancer collection.

Select clinical features of the REMBRANT dataset	Summary of 130 patient cohort before filtering			Summary of 64 patient cohort after filtering		
*Clinical Feature*	*Category*	*Patient count*	%	*Category*	*Patient count*	%
Age range	10–14	1	1%	10–14	1	2%
	15–19	2	2%	15–19	1	2%
	20–24	3	2%	20–24	0	0%
	25–29	4	3%	25–29	3	5%
	30–34	7	5%	30–34	5	8%
	35–39	13	10%	35–39	4	6%
	40–44	7	5%	40–44	3	5%
	45–49	8	6%	45–49	5	8%
	50–54	11	8%	50–54	6	9%
	55–59	6	5%	55–59	3	5%
	60–64	6	5%	60–64	1	2%
	65–69	3	2%	65–69	2	3%
	70–74	6	5%	70–74	3	5%
	75–79	3	2%	75–79	2	3%
	85–89	1	1%	85–89	1	2%
	NA or blank	49	38%	NA or blank	24	38%
Gender	FEMALE	37	28%	FEMALE	16	25%
	MALE	43	33%	MALE	24	38%
	NA or Blank	50	38%	NA or Blank	24	38%
Disease Type	ASTROCYTOMA	47	36%	ASTROCYTOMA	28	44%
	GBM	41	32%	GBM	18	28%
	MIXED	1	1%	OLIGODENDROGLIOMA	12	19%
	OLIGODENDROGLIOMA	22	17%	NA or Blank	6	9%
	UNCLASSIFIED	1	1%			
	NA or Blank	18	14%			

**Fig. 4:**
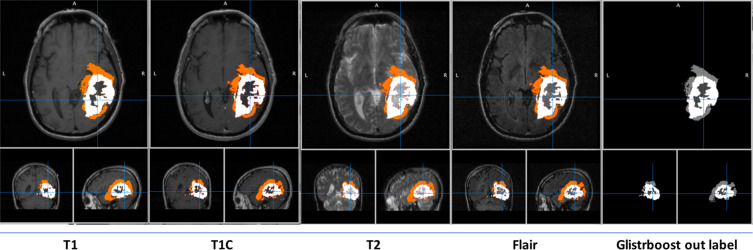
Segmented labels for a brain cancer patient (patient# HF1538) obtained using the GLISTRboost pipeline.

The output files from this pipeline were in the form of NIfTI files https://nifti.nimh.nih.gov. Figure 4 shows the segmented labels for a brain cancer patient obtained using the GLISTRboost pipeline.

### Radiomics analysis

Our Board-Certified radiologist discovered that the BraTumIA algorithm was only effective in the segmentation of one type of cancer, i.e., GBM patients; whereas the GLISTRboost pipeline produced more accurate segmented labels for all the brain cancer sub-types in this data collection. For this reason, we chose the segmented labels from the GLISTRboost pipeline for the radiomics analysis.

Pyradiomics^41^, an open-source python package was used to extract radiomics features from the segmented labels of the MRI brain scans. It included a total of 120 features, which describes various properties related to the medical image pixels, including two- and three-dimensional shape, texture, energy and entropy, size and co-occurrence, gray tone differences and more^41^. Table 3 shows a summary of the different classes of features characterized by pyradiomics^42^. Supplementary File 1 shows the radiomics features extracted from the REMBRANDT segmented labels from the GLISTRboost pipeline.

**Table 3:** Summary of the types of features represented in the pyradiomics numerical output.

Class of Pyradiomics feature	Number of features
First Order Statistics	19
Shape-based (3D)	16
Shape-based (2D)	10
Gray Level Co-occurrence Matrix	24
Gray Level Run Length Matrix	16
Gray Level Size Zone Matrix	16
Neighboring Gray Tone Difference Matrix	5
Gray Level Dependence Matrix	14
Total	120

### Applications

#### Applications for multi-omics analysis

The gene expression and copy number data from this same dataset was made public in 2018 through the publication *Gusev et al*.^16^, and the data made available the NCBI Gene Expression Omnibus (GEO) data repository GSE108476^17^. The medical imaging data in the form of segmented labels, along with numerical output from radiomics will now be made public through this publication. This would allow researchers to integrate gene expression, copy number and medical imaging data from the same set of patients. Such a multi-omics based radiogenomics analyses would allow for research and development of novel biomarkers, and treatment hypotheses for precision medicine.

#### Applications for meta-analysis of brain cancer imaging studies

The GLISTRboost segmentation pipeline used in this paper has been applied to the MRI scans from TCGA brain cancer (TCGA-GBM and TCGA-LGG) patients as demonstrated in the *Bakas et al*.^12^ publication. Since the same GLISTRboost segmentation pipeline was applied to the REMBRANDT and TCGA brain cancer (TCGA-GBM and TCGA-LGG), we can now use them for meta-analyses. For instance, the open source radiomics PyRadiomics tool can be used on both datasets to obtain quantitative radiomics output. This means that these two data collections could be used together in a meta-analysis approach to provide a better sample size for machine learning and AI applications. We believe this is very valuable and enables further biomedical and integrative data analysis. The radiomics output from PyRadiomics from the REMBRANDT; and the TCGA-GBM and TCGA-LGG collections have been made available through this publication as Supplementary File 1 and Supplementary File 2 respectively.

#### Applications for federated learning approaches in brain cancer imaging studies

Another application is the Federated Tumor Segmentation (FeTS) platform^43^ that allows training specific machine learning models by leveraging information gathered from brain cancer datasets residing in collaborating sites without ever exchanging the data. The segmented labels from our REMBRANDT MRI scans are part of this world-wide federation https://www.fets.ai/, and has enabled very large multi-site machine learning models in an effort to accelerate discovery.

### Summary

In this publication, we took the raw MRI scans from the REMBRANDT data collection from public domain, and performed volumetric segmentation to identify various subregions of the brain. Radiomic features were then extracted to represent the MRI scans in numerical format. The gene expression and copy number data from the same Rembrandt dataset was made public in 2018 through the publication *Gusev et al*.^16^, and the data made available the NCBI Gene Expression Omnibus (GEO) data repository GSE108476^17^. This dataset now enables researchers to further translational research using not only the medical image data, but also in conjunction with the genomics and clinical data.

We believe that by making this dataset available to the research community via a public repository provides a unique data science research opportunity to the biomedical and data science research communities. Such combined datasets would provide researchers with a unique opportunity to conduct integrative analysis of quantitative data from medical images, gene expression and copy number changes, alongside clinical outcomes (overall survival) in this large brain cancer study published to date.

## Technical Validation - Radiologist Manual Verification

Our Board-Certified radiologist confirmed that the BraTumIA algorithm was only effective in the segmentation of one type of cancer – GBM patients. This is mentioned in the BraTumIA manual (https://www.nitrc.org/projects/bratumia), and is due to the fact that the morphology is very different for each cancer subtype, and hence the tool worked well only for GBM patients

The radiologist found that the GLISTRboost algorithm was more effective in the segmentation of the various sub-types of brain cancers in this dataset – Astrocytoma, Oligodendroglioma, and GBM. Manual verification and correction were performed on the segmented labeled output files. By using an additional manual *seeding* step which provided sample sub-regions as a reference for the algorithm, the GLISTRboost pipeline was able to overcome morphology and other differences in the various sub-types of brain cancers in this dataset.

**Fig. 5:**
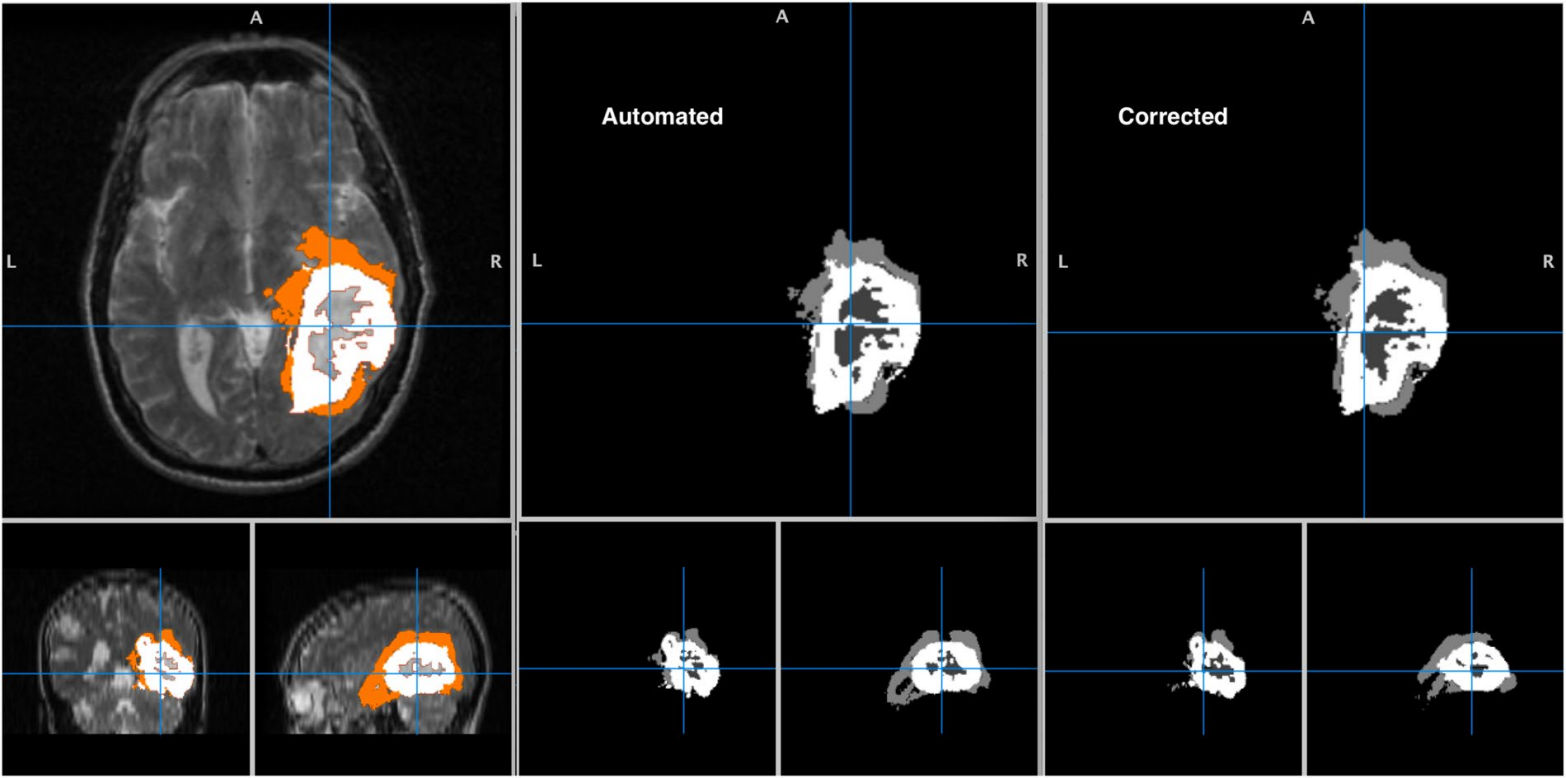
Illustration of how the Radiologist performed manual verification using patient# HF1538 as an example.

This verification and corrections were performed using an MRI viewer software MITK^44^ https://www.mitk.org/. Figure 5 shows an example image of how the manual verification performed.

## Data Records

We first downloaded the pre-operative raw MRI scans from the TCIA imaging archive for 130 patients. After cleaning, MRI scans from 72 patients with complete data from four modalities were chosen for further processing. Two well-known brain cancer segmentation pipelines were applied to the cleaned dataset – BraTumIA^23^ and GLISTRboost^24^. The GLISTRboost^24^ algorithm was top ranked in the International Multimodal Brain Tumor Image Segmentation challenge 2015 (BraTS’15), and the BraTumIA^23^ algorithm was the runner up. After running both the BraTumIA^23^ and GLISTRboost^24^ pipelines, it was discovered that BraTumIA^23^ tool was only effective in the segmentation of one type of cancer – GBM patients. GLISTRboost^24^ pipeline was more effective in the segmentation of the various sub-types of brain cancers in this dataset – Astrocytoma, Oligodendroglioma, and GBM.

The segmented labels from the GLISTRboost^24^ pipeline, along with the manual corrections performed radiologist have been made publicly available through NeuroImaging Tools & Resources Collaboratory (NITRC) repository^19^. The gene expression and copy number data from this same dataset was made public in 2018 through the publication *Gusev et al*.^16^, and the data made available the NCBI Gene Expression Omnibus (GEO) data repository GSE108476^17^. Table 3 shows a high-level summary of the REMBRANDT brain cancer collection.

## Usage Notes

The *Madhavan*^45^ *et al*. publication that originally described the Rembrandt portal and dataset has enabled numerous analyses and has been cited 366 times so far (as of January 2022). The gene expression and copy number data from the REMBRANDT dataset was made public in 2018 through the publication *Gusev et al*.^16^, and the data made available the NCBI Gene Expression Omnibus (GEO) data repository GSE108476^17^ which has been cited 69 times so far (as of January 2022).

In this publication, we took the raw MRI scans from the REMBRANDT data collection and performed volumetric segmentation to identify various subregions of the brain. Radiomic features were then extracted to represent the MRI scans in a quantitative format. This dataset now enables researchers to integrate gene expression, copy number and medical imaging data from the same set of patients. Such a multi-omics based radiogenomics analyses would allow for research and development of novel biomarkers, and treatment hypotheses for precision medicine.

The GLISTRboost segmentation pipeline applied in this manuscript was previously applied to the MRI scans from TCGA brain cancer (TCGA-GBM and TCGA-LGG) patients in *Bakas et al*.^12^ publication. Since imaging data from both REMBRANDT and TCGA brain cancer collection were processed with the same segmentation pipeline, the two datasets can now be used in-conjunction in a meta-analyses study. For example, the TCGA brain cancer dataset could be used as a training set, and the REMBRADNT dataset could be used as an independent testing set in such an analysis. Another example: open source radiomics tool PyRadiomics can be applied to both datasets to obtain quantitative radiomics output. Such a meta-analysis approach can provide a better sample size for machine learning and AI applications. We believe this would be very valuable and enables further biomedical and integrative data analysis. The radiomics output from PyRadiomics from the REMBRANDT; and the TCGA-GBM & TCGA-LGG collections have been made available through this publication as Supplementary File 1 and Supplementary File 2 respectively.

Another application is the Federated Tumor Segmentation (FeTS) platform^43^ that allows training specific machine learning models by leveraging information gathered from brain cancer datasets residing in collaborating sites without ever exchanging the data^43^. The segmented labels from our REMBRANDT MRI scans are part of this world-wide federation https://www.fets.ai/. Such a federated model has enabled very large multi-site machine learning models in an effort to accelerate discovery, and build new advanced machine learning models.

In summary, we believe that by making this dataset available to the research community via a public repository provides a unique data science research opportunity to the biomedical and data science research communities. Such combined datasets would provide researchers with a unique opportunity to conduct integrative analysis of numerical data from medical images, gene expression and copy number changes, alongside clinical outcomes (overall survival) in this large brain cancer study.

## Data Privacy

The segmented medical images generated in this manuscript and made public via NITRC are skull stripped and hence do not contain identifiable information.

## Supplementary information

Supplementary File 1:

Supplementary File 2

## Data Availability

The methods and tools applied in this paper use open-source tools detailed in respective publications *Bakas et al*.^12^ publication. The python code for extracting PyRadiomics features from Rembrandt and the TCGA segmented data (Supplementary File 1 and 2 respectively) is provided here. https://github.com/ICBI/rembrandt-mri.
