# Author Correction: Enhancing the REMBRANDT MRI collection with expert segmentation labels and quantitative radiomic features

**DOI:** 10.1038/s41597-022-01518-9

**Published:** 2022-07-07

**Authors:** Anousheh Sayah, Camelia Bencheqroun, Krithika Bhuvaneshwar, Anas Belouali, Spyridon Bakas, Chiharu Sako, Christos Davatzikos, Adil Alaoui, Subha Madhavan, Yuriy Gusev

**Affiliations:** 1 Medstar Georgetown University Hospital, Washington, DC USA; 2 Innovation Center for Biomedical Informatics (ICBI), Georgetown University, Washington, DC USA; 3 Center for Biomedical Image Computing and Analytics (CBICA), University of Pennsylvania, Philadelphia, PA USA

**Keywords:** Data publication and archiving, Data processing, Image processing

Correction to: *Scientific Data* 10.1038/s41597-022-01415-1, published online 14 June 2022

In this article the affiliation details for Anousheh Sayah were incorrectly given as ‘Innovation Center for Biomedical Informatics (ICBI), Georgetown University, Washington, DC, USA’ but should have been ‘Medstar Georgetown University Hospital, Washington, DC, USA’.

In this article the affiliation details for Camelia Bencheqroun were incorrectly given as ‘Medstar Georgetown University Hospital, Washington, DC, USA’ but should have been ‘Innovation Center for Biomedical Informatics (ICBI), Georgetown University, Washington, DC, USA’.

